# Perivascular Mural Cells of the Mouse Choroid Demonstrate Morphological Diversity That Is Correlated to Vasoregulatory Function

**DOI:** 10.1371/journal.pone.0053386

**Published:** 2013-01-04

**Authors:** Audrey B. Condren, Anil Kumar, Pradeep Mettu, Katharine J. Liang, Lian Zhao, Jen-yue Tsai, Robert N. Fariss, Wai T. Wong

**Affiliations:** 1 Unit on Neuron-Glia Interactions in Retinal Disease, National Eye institute, National Institutes of Health, Bethesda, Maryland, United States of America; 2 Biological Imaging Core, National Eye institute, National Institutes of Health, Bethesda, Maryland, United States of America; Institut de la Vision, France

## Abstract

**Objective:**

Perivascular mural cells of the choroid have been implicated in physiological functioning as well as in retinal disease pathogenesis. However details regarding their form and function are not well understood. We aim to characterize choroidal mural cells in the adult mouse choroid in terms of their distribution and morphology, and correlate these to their contractile behavior.

**Methods:**

Sclerochoroidal flat-mounted explants were prepared from albino transgenic mice in which the α-smooth muscle actin (α-SMA) promoter drives the expression of green fluorescent protein (GFP). α-SMA-expressing smooth muscle cells and pericytes in the living choroid were thereby rendered fluorescent and imaged with confocal microscopy and live-cell imaging *in situ.*

**Results:**

Choroidal perivascular mural cells demonstrate significant diversity in terms of their distribution and morphology at different levels of the vasculature. They range from densely-packed circumferentially-oriented cells that provide complete vascular coverage in primary arteries to widely-spaced stellate-shaped cells that are distributed sparsely over terminal arterioles. Mural cells at each level are immunopositive for contractile proteins α-SMA and desmin and demonstrate vasoconstrictory contractile movements in response to endothelin-1 and the calcium ionophore, A23187, and vasodilation in response to the calcium chelator, BAPTA. The prominence of vasoregulatory contractile responses varies with mural cell morphology and density, and is greater in vessels with dense coverage of mural cells with circumferential cellular morphologies. In the choriocapillaris, pericytes demonstrate a sparse, horizontal distribution and are selectively distributed only to the scleral surface of the choriocapillaris.

**Conclusions:**

Diversity and regional specialization of perivascular mural cells may subserve varying requirements for vasoregulation in the choroid. The model of the α-SMA-GFP transgenic albino mouse provides a useful and intact system for the morphological and functional study of choroidal mural cells.

## Introduction

The choroid of the eye consists of a specialized vascular bed that supplies the outer retina. It provides metabolic support to the specialized functions of the photoreceptors and retinal pigment epithelial (RPE) cells and mediates thermoregulation, eye growth, and emmetropization [1]. Significantly, changes at the level of the choroid feature prominently in the pathology of multiple retinal diseases, particularly age-related macular degeneration (AMD) [2], [3]. Understanding the physiological functioning of the choroid and how it undergoes pathological transformations may be significant in considering choroid-directed therapies in the prevention and treatment of retinal disorders [4]–[6].

The normal physiological functioning of the choroid, as well as its pathological changein disease, have been associated with the perivascular component of the choroid. Perivascular mural cells located in close proximity to endothelial cells of choroidal vessels are thought to play a role in regulating blood flow and hence controlling metabolic supply to the outer retina. Inadequacy of this metabolic supply (otherwise termed “choroidal insufficiency”) has been hypothesized to be present in retinal diseases such as AMD [7]–[10] and diabetic retinopathy [11]–[13]. Perivascular cells around choroidal vessels have also been hypothesized to regulate the stability of choroidal vasculature [6], [14], and are relevant to the formation of choroidal neovascularization (CNV) [15]–[17], the aberrant vascular growth responsible for vision loss in the exudative form of AMD, and also to the resistance of CNV to anti-vascular endothelial growth factor (VEGF) therapies [18], [19].

Despite these functional significances, the perivascular compartment of the choroid, consisting of perivascular smooth muscle and pericytes, has not been previously characterized in detail and questions concerning their function and localization remain. Although perivascular cells are likely to contribute to the regulation of choroidal blood flow, how they do so at different levels of the choroidal vasculature is unclear [20]. While pericytes in vessels of the retina have been demonstrated to exhibit contractile properties *in situ* [21], a similar demonstration has not been made for mural cells in the choroid. *In situ* studies of perivascular cells in the choroid have been limited by the relative optical inaccessibility; pigmentation in the choroid and overlying RPE can obscure their visualization in tissue explants, and immunohistochemical markers have not consistently labeled these cells in their entirety to reveal full morphological features.

In the current study, we have employed an albino transgenic mouse model which expresses green fluorescent protein (GFP) specifically in α-smooth muscle actin expressing cells. This system allowed us to image the distribution and morphology of perivascular cells of the choroid. We prepared intact sclerochoroidal explants and used live-cell confocal imaging techniques to observe and analyze contractile movements in these cells. Our findings here reveal that perivascular mural cells consisting of smooth muscle cells and pericytes demonstrate a graded diversity in their distribution and morphology at each level of the choroidal vasculature and that they demonstrate calcium-dependent contractile movements well-suited for vasoregulation. We also found that perivascular cell density and morphology correlated with contractile capability, indicating that mural cell diversification and patterning in the choroid may subserve the need for vasoregulatory function at each level of the choroid. These observations reflect functionally significant regional specializations of perivascular mural cells in the choroid and shed light on mechanisms potentially relevant to abnormal choroidal flow and vessel destabilization in retinal diseases.

## Materials and Methods

### Experimental Animals

Alpha-smooth muscle actin (α-SMA) transgenic mice were produced in the Transgenic Mice Facility at the National Eye Institute, NIH, on a C57BL/6 strain background. These transgenic mice express green fluorescent protein (GFP) under the control of the α-SMA promoter, resulting in the specific expression of GFP in both vascular and non-vascular smooth muscle cells [22]. In ocular structures, GFP was found expressed in mural cells of the retinal vasculature [23]. In order to visualize mural cells in the mouse choroid in sclerochoroidal flat-mounts, we bred α-SMA transgenic mice with wild type BALB/c albino mice (Charles River, Wilmington, MA). Progeny from the F1 generation were interbred, and F2 progeny which were albino in coat coloration and expressed the α-SMA-GFP transgene were selected and interbred. All animals were bred and housed in a National Institutes of Health animal facility. Experiments were conducted according to protocols approved by the local Institutional Animal Care and Use Committee and adhered to the ARVO Statement for the Use of Animals in Ophthalmic and Vision Research.

### Direct Labeling and Visualization of Choroidal Vessels in Sclerochoroidal Flat-mounted Tissue

Blood vessels in the choroid are directly labeled by cardiac perfusion of an aqueous solution containing 1,1′-dioctadecyl-3,3,3′,3′-tetramethylindocarbocyanine perchlorate (DiI, D-282, Invitrogen/Molecular Probes, Carlsbad, CA), a lipophilic dye that labels endothelial cell membranes by incorporation as previously described [24]. Briefly, albino α-SMA transgenic mice were euthanized by carbon dioxide inhalation and the thoracic cavity opened to expose the heart. A volume of 100 µl of DiI stock solution (6 mg/ml in 100% ethanol) was dissolved in 5 ml of diluent comprising of PBS and 5% glucose in a 1∶4 ratio and the resulting solution applied via cardiac perfusion at a rate of 1–2 ml/min. This was immediately followed by the perfusion of 5–10 ml of PBS and 5–10 mls of 4% paraformaldehyde at a rate of 1–2 ml/min. The eyes were harvested and the retinal pigment epithelium (RPE)-sclera-choroid complex dissected free of other ocular structures. These flat-mount preparations were mounted on glass slides in mounting medium (Fluoromount; Sigma, St. Louis, MO) with the RPE cell layer uppermost. This technique enabled the visualization of GFP-labeled choroidal perivascular mural cells in the context of DiI-labeled endothelial cells in an *in situ* choroidal flat-mounted preparation.

### Immunohistochemistry

After fixation, sclerochoroidal flatmounts were washed thrice in 1× PBS, transferred into PBS containing 1% Triton-X100 (Sigma) for 1 hour at room temperature, and then incubated in blocking buffer (Roche, Indianopolis, IN, USA) for 30 minutes on a shaker. Primary antibodies to the following antigens were used: laminin (1∶25; Sigma) for staining vascular cell basement membranes, α-SMA (1∶200, Sigma), NG2 (1∶200; Upstate Cell Signaling Solutions, CA) and anti-desmin (1∶20; Cat # 10519, MP Biomedicals, Solon, OH) for labeling choroidal smooth muscle cells and pericytes. Secondary antibodies, conjugated to Alexa-488, Alexa-568, or Alexa-633 (Invitrogen), were added at a 1∶200 dilution and incubated for 1–2 hours. 4′,6-diamidino-2-phenylindole (DAPI, Molecular Probes/Invitrogen, Cat# D1306) was used to label cellular nuclei. Antibodies were diluted in a solution of 0.5% bovine serum albumin (BSA), 2% normal goat serum, 0.2% Tween-20, 0.05% sodium azide, 1XPBS (all from Sigma).

### *Ex vivo* Time-lapse Imaging of Perivascular Mural Cells in the Choroid

α-SMA-GFP transgenic mice were euthanized and immediately enucleated. Sclerochoroidal explants were acutely isolated from the eyecups, flat-mounted with the RPE cell layer uppermost on filter paper (HABP045; Millipore, Billerica, MA) and placed in an oxygenated Ringer’s solution comprising of: 125 mM NaCl, 5 mM KCl, 1.5 mM CaCl_2_, 0.75 mM MgCl_2_/6H_2_O, 1.25 mM NaH_2_PO_4_, 10 mM D-glucose, 20 mM HEPES (pH 7.35–7.45) (all reagents from Sigma). For live imaging of choroidal vessels, explants were transferred to a microscope stage-mounted temperature-controlled imaging chamber (Bioptechs, Butler, PA) maintained at 32°C. Oxygenated Ringer’s solution was continuously superfused through the chamber during the recording. Live GFP-labeled perivascular mural cells in the choroid could be clearly visualized through the overlying RPE-cell layer using a confocal microscope (SP2; Leica, Exton, PA) and a 40× (0.80 numerical aperture) immersion objective. Z-series image stacks that traversed the thickness of the choroid were collected at regular time intervals Maximum projections of the image stacks in the Z-direction were created for morphological and motility analyses. During the imaging session, the following agonists in Ringer’s solution was superfused into the recording chamber at specified time-points for 15–30 minutes, followed by a washout period of 40–60 minutes with Ringer’s solution: Endothelin-1 (10 nM, 7764, Sigma), calcium ionophore, A23187 (5–10 µM, C7522, Sigma),and calcium chelator, BAPTA (10 µM, A4926, Sigma).

### Quantitative Analysis of Mural Cell Distribution and Contractility

For morphological analysis of choroidal vessels, two-dimensional (2D) representations of GFP-positive murals cells were created from maximum intensity projections of image stacks completely traversing choroidal vessels. Owing to the circumferential distribution and orientation of the mural cells, the dimensions of choroidal vessels were well-delineated and could be measured using image analysis software (ImageJ, National Institutes of Health, Bethesda, MD). Vascular diameter was defined as the distance between GFP-positive mural cells on opposing luminal walls measured in the plane orthogonal to the longitudinal axis of the vessel. Vascular cross-sectional area was calculated from vascular diameter, modeling the vessel lumen as a geometric cylinder. The linear density of mural cells was calculated as the number of mural cells identified along a 100 µm-long section of the vessel.

### Statistical Analysis

Results were analyzed by non-parametric ANOVA tests and are expressed as mean ± SEM.

## Results

### Distribution of α-SMA-GFP Perivascular Cells in the Mouse Choroid

**Figure 1:**
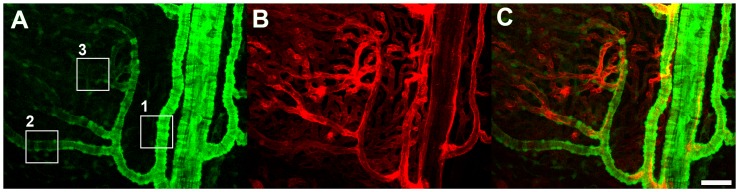
Distribution of green fluorescent protein (GFP)-labeled cells in the choroid of an adult albino α-SMA-GFP transgenic mouse. (**A**) Choroidal vasculature as visualized by confocal microscopy through the RPE cell layer in a sclerochoroidal flat-mount. GFP-positive perivascular mural cells are present in choroidal arteries (box 1), smaller secondary arterioles (box 2), and branching precapillary arterioles (box 3). (**B**) Labeling of the endothelial layer throughout the choroidal vasculature was achieved with systemic perfusion of the lipophilic dye DiI, which becomes incorporated into endothelial cell membranes. (**C**) Superposition of the GFP and DiI signals demonstrates the perivascular nature of GFP-positive cells in the choroid. Scale bar = 50 µm.

Sclerochoroidal flat-mounts were prepared from albino α-SMA-GFP transgenic mice, mounted on glass slides with the RPE layer uppermost, and imaged with confocal microscopy. Owing to the absence of pigmentation in the RPE and the choroid, and the lack of GFP expression in RPE cells, α-SMA-positive perivascular cells were clearly observed *in situ* throughout the thickness of the choroid beneath the intact RPE layer. GFP-associated fluorescence was found distributed throughout the cytoplasm of labelled cells, clearly revealing the morphological features of α-SMA- expressing cells at different levels of the vascular system (Fig. 1A). The perivascular nature of GFP-positive cells was confirmed by the intravascular perfusion of the fluorescent lipophilic dye, DiI, which was incorporated into the cell membrane of endothelial cells, marking the vascular lumina of the entire choroidal vascular tree (Fig. 1B). GFP-positive cells were consistently observed in a perivascular location, surrounding choroidal arteries, arterioles (Fig. 1C), and the choriocapillaris.

**Figure 2:**
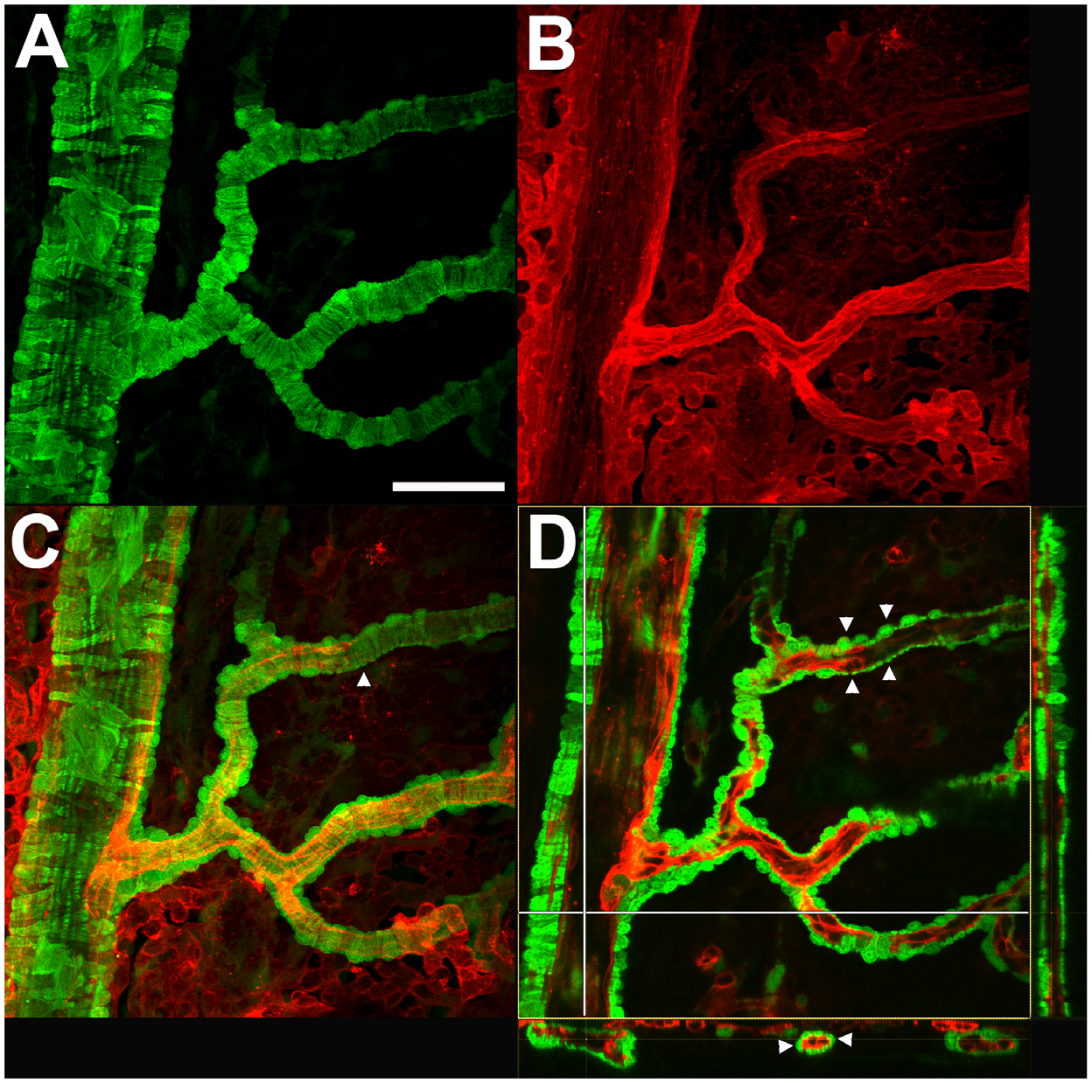
Morphology and distribution of GFP-positive perivascular mural cells of primary choroidal arteries (Type 1 vessels). Perivascular GFP-positive mural cells (**A**) are densely distributed along the length of DiI-perfused large main choroidal arteries (**B**). Superposition of a z-stack of confocal images for GFP- and DiI-derived signals (**C**) demonstrates that perivascular mural cells completely envelop the vessel walls. GFP-positive striations of smooth muscle mural cells (*arrowhead, C*) are visible around vessel walls, indicating the presence of encircling processes that wrap around vessels in a continuous manner. A single frame confocal image through large main choroidal arteries (**D**) demonstrates that individual mural cells possess rounded protuberant nuclei and long circumferential processes (*upper arrowheads*). Mural cells and their processes provide a near complete coverage of the choroidal artery walls (*arrowheads in lower cross-sectional panel*) with no significant gaps between neighboring mural cell processes. Scale bar = 50 µm.

The morphology, distribution, and density of α-SMA-expressing mural cells were examined at various levels of the choroidal arterial tree in this preparation. At the level of the primary and secondary choroidal arteries, perivascular mural cells, likely corresponding to smooth muscle cells, have (1) a typical protuberant ovoid soma which is directed away from the luminal surface, and (2) circumferential processes that encircle the circumference of the artery giving rise to a banded or striated appearance (Fig.2A–C). At this level, these mural cells are tightly packed at a high density, with their processes closely juxtaposed alongside to each other as to provide a near complete coverage of the mural surface with very few intervening spaces between cellular processes (Fig. 2D). For the descriptive purposes in this paper, we have termed choroidal vessels with complete coverage by mural cells with banded morphologies as Type 1 vessels.

**Figure 3:**
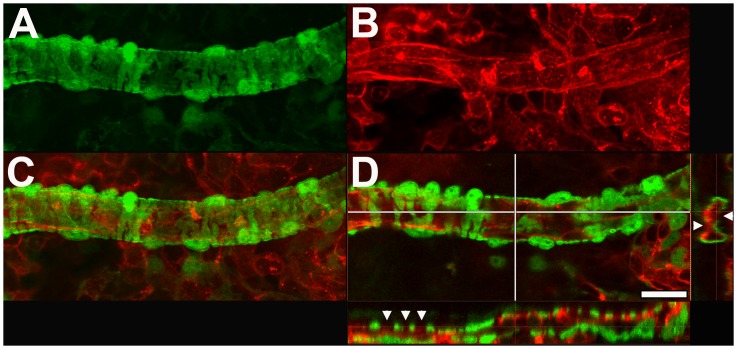
Morphology and distribution of GFP-positive perivascular mural cells of secondary choroidal arterioles (Type 2 vessels). Perivascular GFP-positive mural cells (**A**) are distributed along the length of DiI-perfused arterioles (**B**). Superposition of a z-stack of confocal images for GFP- and DiI-derived signals (**C**) demonstrates that each mural cell consist of a protuberant soma and encircling circumferential processes. A single frame confocal image through an arteriole (**D**) demonstrates that vessel walls are not completely covered by these GFP-positive mural cells, and intervening gaps are present between adjacent cellular processes (*arrowheads* in cross-sectional panels showing longitudinal (*lower*) and transverse (*right*) sections*).* Scale bar  = 20 µm.

At the level of the secondary choroidal arterioles, α-SMA-expressing mural cells demonstrate similar morphological features of a protuberant soma and circumferential processes (Fig. 3A–C); however, these mural cells are distributed along the vessels at a lower density and have more widely spaced processes. As a result, perivascular coverage by mural cells is incomplete, with intervening spaces appearing between neighboring cells and between parallel processes of the same cell (Fig. 3D). We termed these choroidal arterioles, which are incompletely covered with mural cells with banded morphologies, as Type 2 vessels.

**Figure 4:**
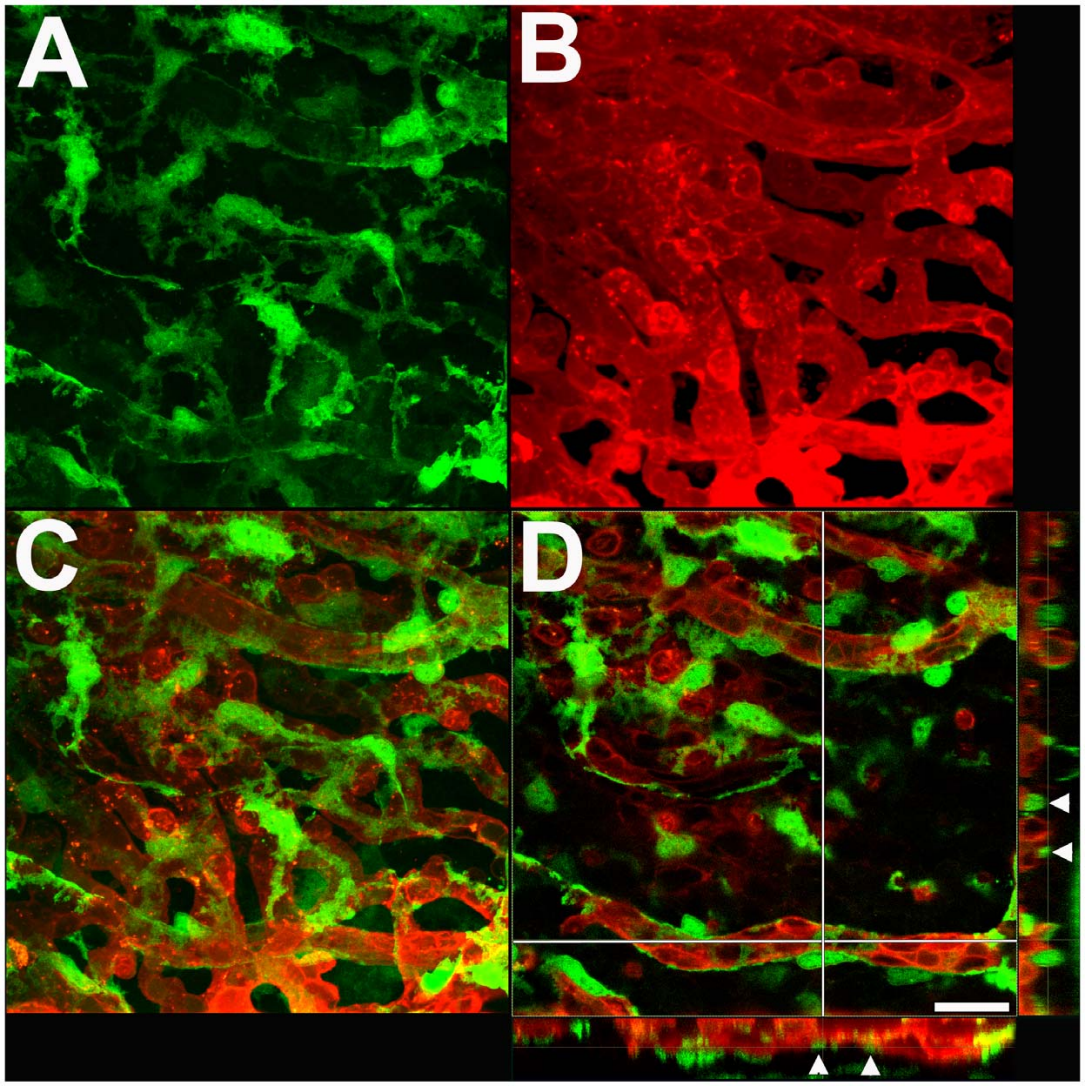
Morphology and distribution of GFP-positive perivascular mural cells of precapillary arterioles (Type 3 vessels). Ramified and stellate-shaped GFP-positive mural cells with thin, branching processes (**A**) are distributed among DiI-perfused precapillary arterioles (**B**). Superposition of a z-stack of confocal images for GFP- and DiI-derived signals (**C**) demonstrates the perivascular position of GFP-positive mural cells. Mural cells are spaced widely along the vessel walls with their processes sparsely distributed on the vessel surface, and are loosely oriented in longitudinal and circumferential directions along the vessels. A single frame confocal image through an arteriole (**D**) demonstrates that only a small fraction of the surface area of vessel is covered by the thin branching mural cell processes (*arrowheads*). Scale bar = 20 µm.

More distally in the choroidal vascular tree at the level of the precapillary arteriole, α-SMA-expressing mural cells progressively transition away from a banded, circumferential morphology towards a stellate shape with ramified tapering processes (Fig. 4A). These cells maintain their perivascular location with somata and processes closely juxtaposed with the vascular endothelium (Fig. 4B–C). These stellate cells are widely spaced, providing a low and highly incomplete coverage of the perivascular surface (Fig. 4D). We termed these precapillary choroidal arterioles with these features as Type 3 vessels.

**Figure 5:**
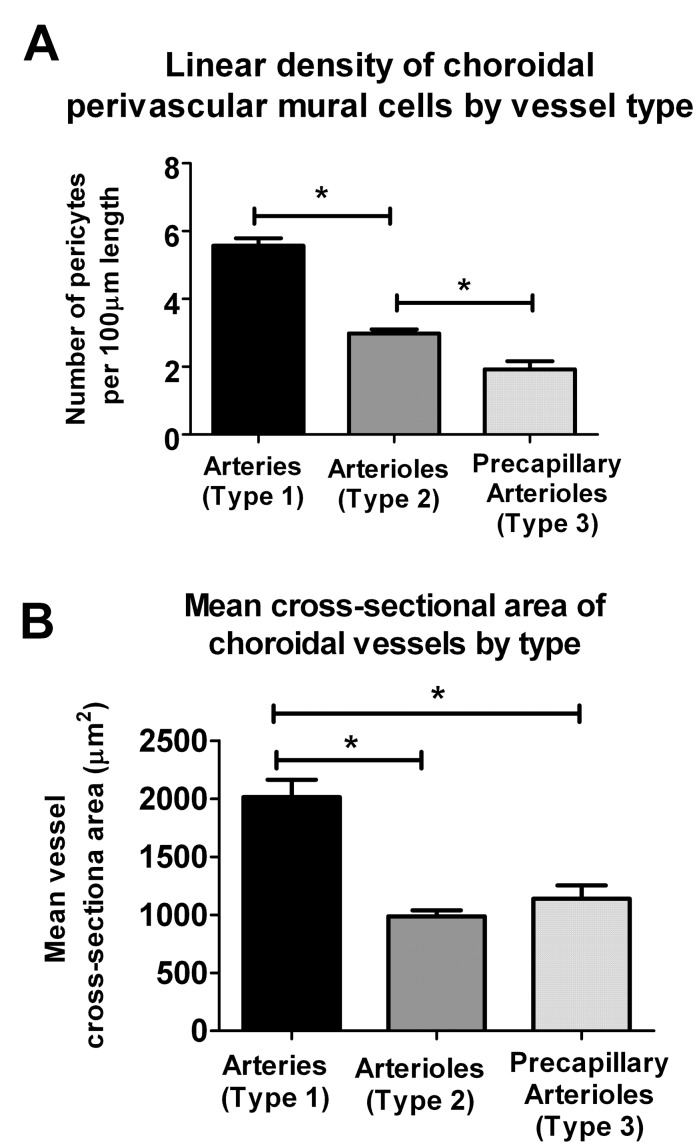
Quantitative comparison of choroidal arteries (Type 1), choroidal arterioles (Type 2), and choroidal precapillary arterioles (Type 3). (**A**) Quantitation of the linear density of perivascular mural cells along choroidal vessels at different levels of the vascular system. Density of mural cells is significantly higher in arteries (type 1 vessels) relative to arterioles (type 2 vessels), which are in turn significantly higher than precapillary arterioles (type 3 vessels) (* indicates p<0.05 on 1-way ANOVA using the Kruskal-Wallis test, with the Dunn’s multiple comparison test, n≥13 measurements from ≥3 biological replicates). (**B**) Mean cross-sectional area of choroidal vessels by type, as calculated from vessel diameter measurements, estimating vessels to have a cylindrical geometry. Cross-sectional areas of arteries (Type 1) were significantly greater than those of arterioles (Type 2 and Type 3) (* indicates p<0.05, 1-way ANOVA using the Kruskal-Wallis test, with the Dunn’s multiple comparison test,n≥51 measurements from ≥3 biological replicates).

The linear density of α-SMA-expressing mural cells was measured and compared between Type 1, 2, and 3 mural vessels (Fig. 5A). Type 1 choroidal arteries have significantly more perivascular cells per unit length than Type 2 secondary arterioles, which in turn have a higher linear density than Type 3 precapillary arterioles. Type 1 arteries have significantly larger cross-sectional diameters than Type 2 or Type 3 vessels (Fig. 5B).

**Figure 6:**
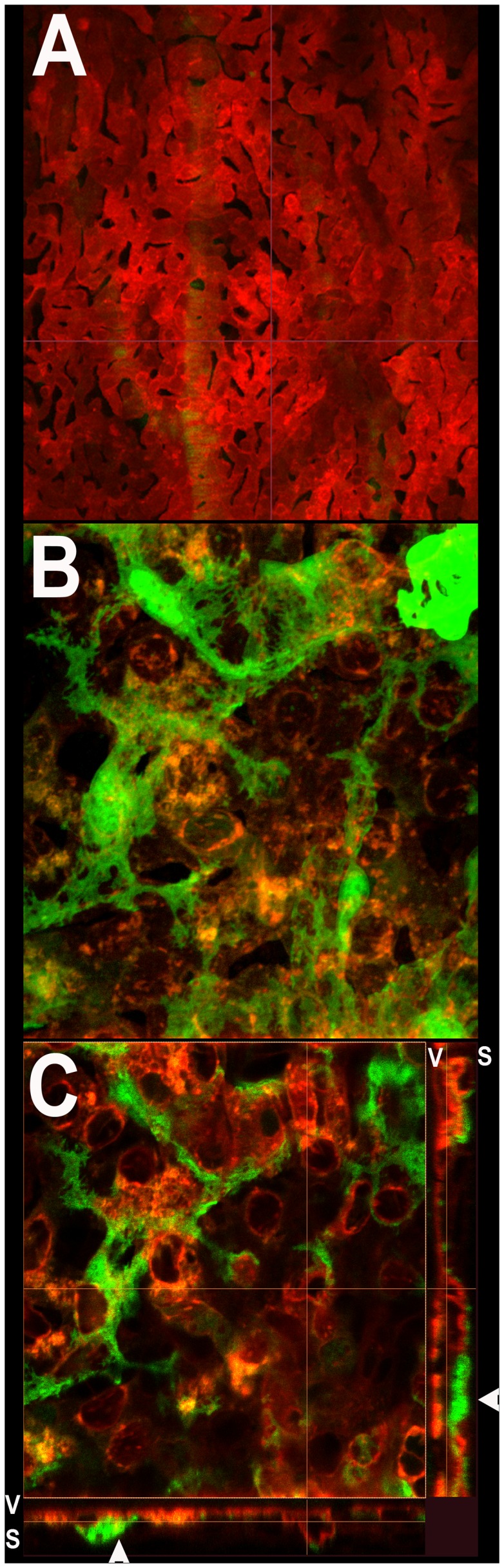
Morphology and distribution of GFP-positive perivascular mural cells in the choriocapillaris. (**A**) Confocal microscopic image of the vitreal surface of choriocapillaris adjacent to the Bruch’s membrane beneath the RPE cell layer. No GFP-positive mural cells are visible on the vitreal surface of the choriocapillaris. (**B**) High magnification maximal projection confocal image of the choriocapillaris as imaged on the scleral surface. Stellate GFP-positive cells with branching processes are found distributed horizontally on the scleral surface of the choriocapillaris in continuous contact with the vascular lobules. (**C**) High magnification single confocal image from the z-stack of images in (B) showing the close apposition of mural cell processes with individual vascular lobules of the choriocapillaris. Orthogonal views of the confocal z-stack (*below* and *right*) demonstrate that mural cell somata (arrowheads) and processes are present on the scleral (S) side of the choriocapillaris but absent on the vitreal (V) side.

At the level of the choriocapillaris, α-SMA-expressing mural cells are notably absent from vitreal surface of the capillary network located adjacent to the Bruch’s membrane (Fig. 6A). Stellate shaped α-SMA-expressing cells are however found distributed sparsely on the scleral surface of the choriocapillaris (Fig. 6B). This polarized distribution of α-SMA-expressing cells is illustrated in a 3- dimensional reconstruction of a confocal z-stack of images taken through the choriocapillaris (Movie S1). The mural cells on the scleral surface of the choriocapillaris have a flattened, horizontal morphology with their somata located in a plane just scleral to and in contact with the choriocapillaris vascular lobules (Fig. 6C, inset below). These cells have fine, symmetrically oriented processes that are distributed across and in between adjacent vascular lobules in the perivascular space.

### Immunohistochemical Characterization of α-SMA-expressing Choroidal Mural Cells

**Figure 7:**
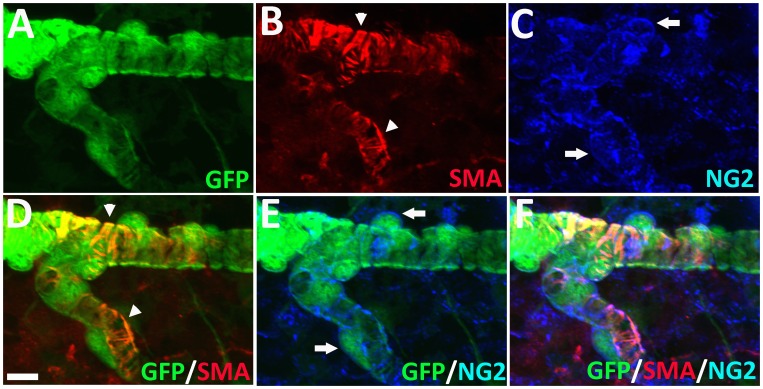
GFP-positive perivascular mural cells in the choroid are immunopositive for α-smooth musle actin (α-SMA) and NG2. GFP-positive mural cells surrounding a Type 2 choroidal arteriole (**A**) were also immunopositive for α-SMA (**B**), visible as banded striations (*arrowheads*) colocalizing with mural cell circumferential cellular processes (**D**). Mural cells were also immunpositive for NG2 (**C**) which was present on the external cell membrane around the somata (*arrows*) and processes (**E, F**). Scale bar = 25 µm.

**Figure 8:**
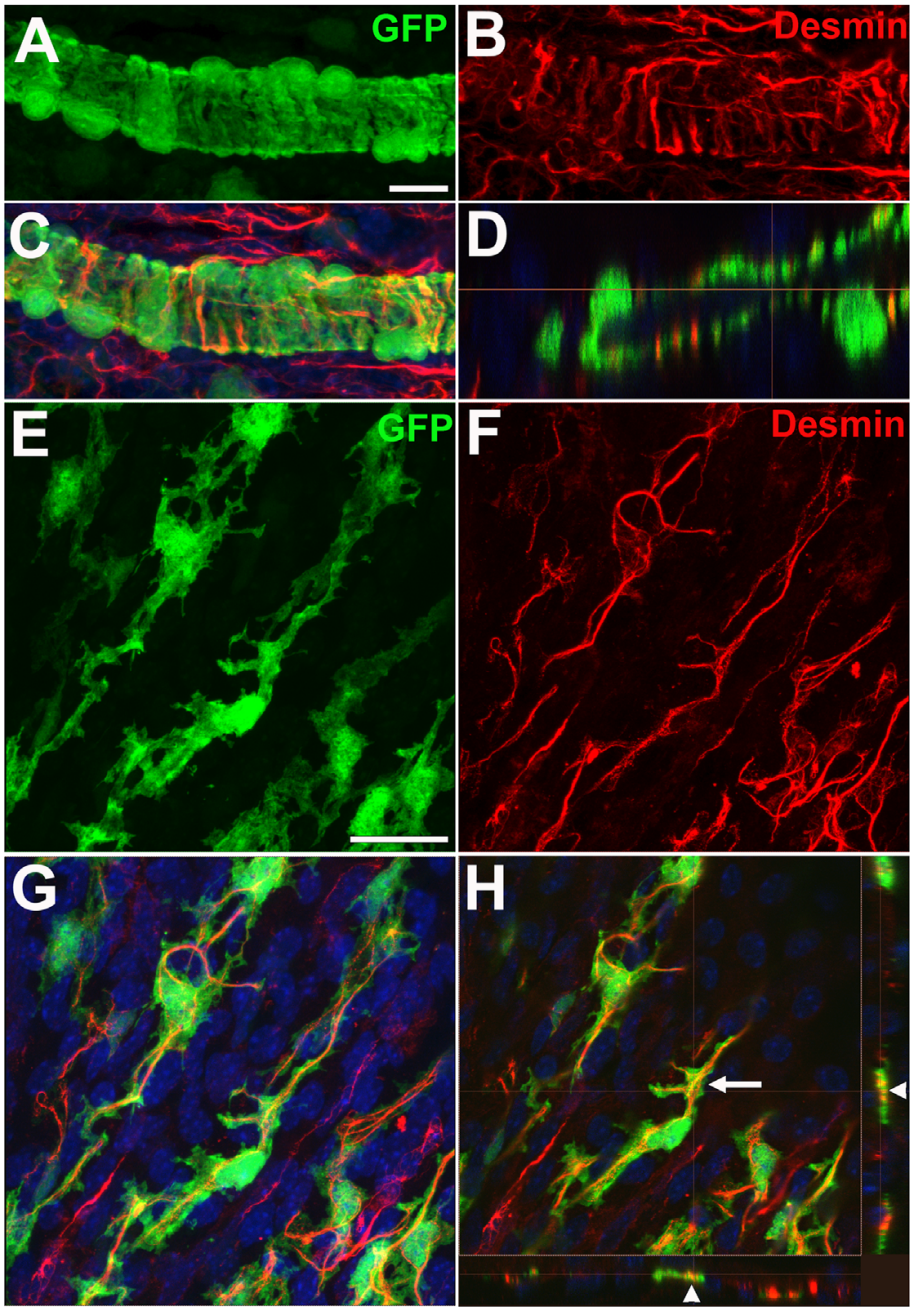
Desmin is expressed in GFP-positive perivascular mural cells in choroidal arterioles. (**A–D**) Secondary choroidal arterioles (Type 2 vessels) with circumferential perivascular GFP-positive mural cells (A) were immunopositive for desmin, an intermediate filament protein (B), which colocalized with the circumferential mural cell processes (C). A confocal section in the longitudinal plane of the arteriole (D) demonstrates that desmin immunopositivity in mural cells has a banded appearance owing to its localization within circumferential mural cell processes (*arrowheads*). Scale bar  = 10 µm. (**E–H**) Precapillary arterioles (Type 3 vessels) with stellate-shaped GFP-positive mural cells (E) were also immunopositive for desmin (F) and had a branching, rather than banded, appearance. Superposition with the GFP-positive mural cells (G) also demonstrated a colocalization to mural cell processes as evidenced in orthogonal confocal images (*arrowheads*) in (H). Scale bar = 25 µm.

We further characterized GFP-labeled perivascular mural cells in the choroid by immunohistochemical staining for contractile proteins and markers found in smooth muscle cells and pericytes. As α-SMA has been previously detected in both smooth muscle cells and pericytes in various organs [25], we similarly found that GFP-labeled choroidal mural cells stained positively with an antibody to α-SMA. Unlike the distribution of GFP which was present uniformly throughout the cytoplasm, α-SMA staining was primarily present within the mural cell processes and appeared as striations running parallel to length of the process (Fig. 7A,B,D). α-SMA staining was notably absent in the cell somata. We found that GFP-labeled mural cells were also positive for NG2, a pericyte marker; labeling was observed on the cell membrane surrouding both the somata and cellular processes (Fig. 7C,E). Immunopositivity for desmin, another contractile protein, was also found to colocalize with the processes of mural cells (Fig. 8). In type 1 and 2 vessels where the mural cells have banded, circumferential processes, desmin labeling appeared as circumferential striations that ran along the lengths of each process (Fig. 8A–D). In type 3 vessels where the mural cells appeared stellate with branching processes, desmin labeling acquired a branching pattern that colocalized with cellular processes in a matching geometry (Fig. 8E–H). Immunolabeling with laminin, a basement membrane marker, revealed that GFP-positive mural cells were individually enclosed in a basement membrane layer (Fig. S1).

### Control of Choroidal Vascular Diameter by Contractility in Choroidal Mural Cells

**Figure 9:**
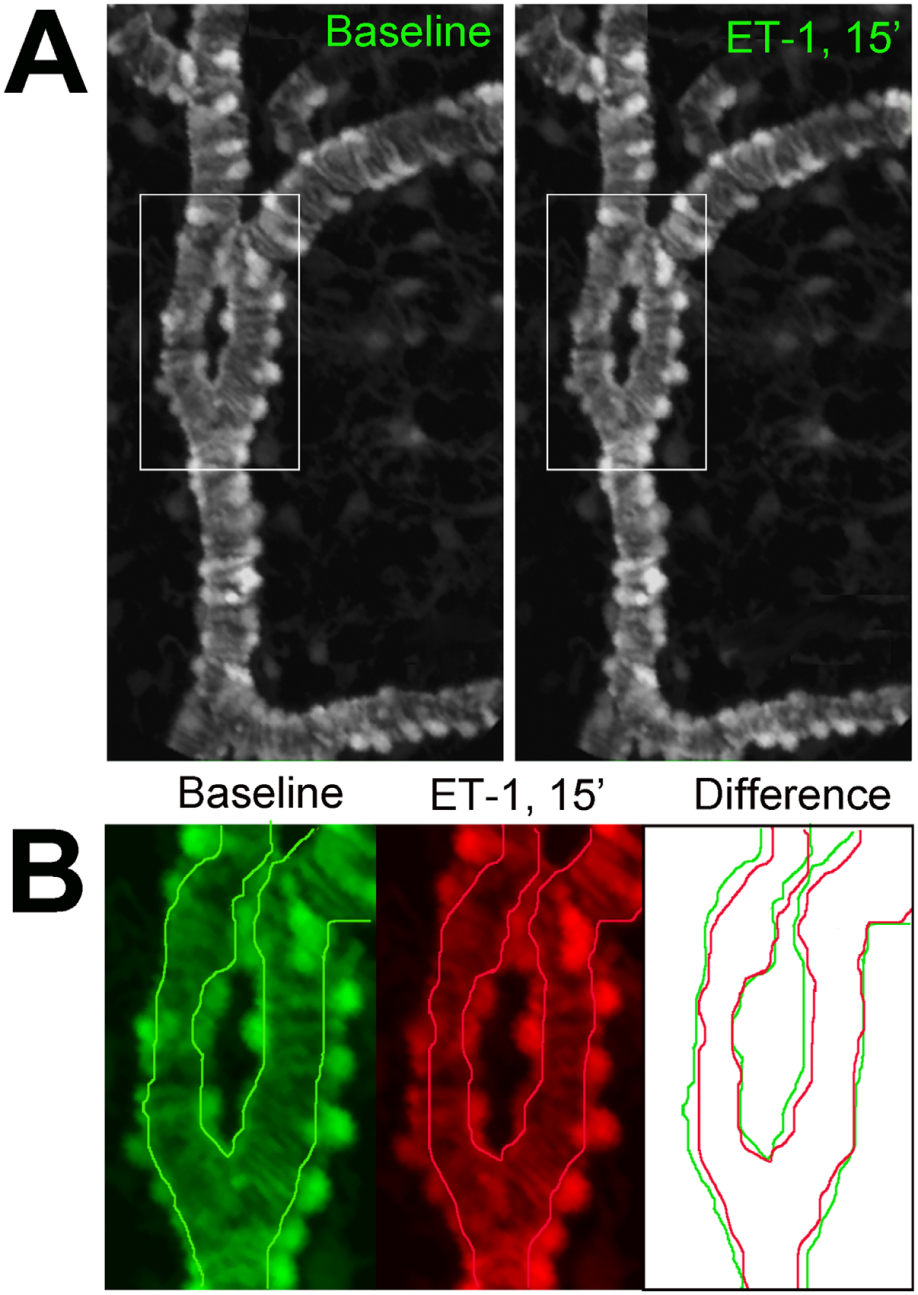
Choroidal vessels demonstrate constriction in response to application of endothelin-1 (ET-1). An example of a choroidal arteriole (type 2) vessel (**A**) showing vascular constriction from baseline conditions (*left*) and 15 minutes after application of endothelin-1 (10 nM) (*right*) (see Move S2). (**B**) Superposition of the vascular lumen profiles from the inset in (A) shows reduction of vessel diameter in response to endothelin.

The close juxtaposition of GFP-labeled mural cells to choroidal vessels at multiple levels of the choroidal vasculature, the expression of contractile proteins in mural cells, and the localization and alignment of these contractile proteins to the longitudinal aspect of cellular processes, indicate that choroidal mural cells may possess contractility and have the ability to regulate vascular diameter, and therefore bloodflow. The vital expression of GFP in our experimental system rendered choroidal mural cells visible in a living, intact, *ex vivo* and *in situ* preparation that allowed mural cell contractility to be dynamically monitored. We assessed the ability of mural cells to contract and influence choroidal diameter using time-lapse, confocal imaging in sclerochoroidal flat-mounted explants. Tissue explants were acutely isolated from experimental animals and maintained in a temperature-controlled, stage-mounted, chamber through which oxygenated Ringer’s solution was perfused. Choroidal mural cells were imaged before, during, and following the superfusion of the vasoconstriction agent, endothelin-1 (ET-1) into the recording chamber. We observed that ET-1 rapidly induced the contractility in mural cells (Fig. 9A) in Type 1, 2, and 3 vessels within the first 5–15 minutes following the perfusion of ET-1. These contractile movements resulted in a sustained constriction in vessel diameter (Fig. 9B) that was only partially reversed 60 minutes following the washout of ET1 (Movie S2). Contractility in mural cells was likely mediated by increased intracellular calcium, as a similar vasoconstriction was induced by the superfusion of A23187, a calcium ionophore (Movie S3). Conversely, superfusion of BAPTA (10 µM), a calcium chelator, induced vasodilation of choroidal vessels (Movie S4).

**Figure 10:**
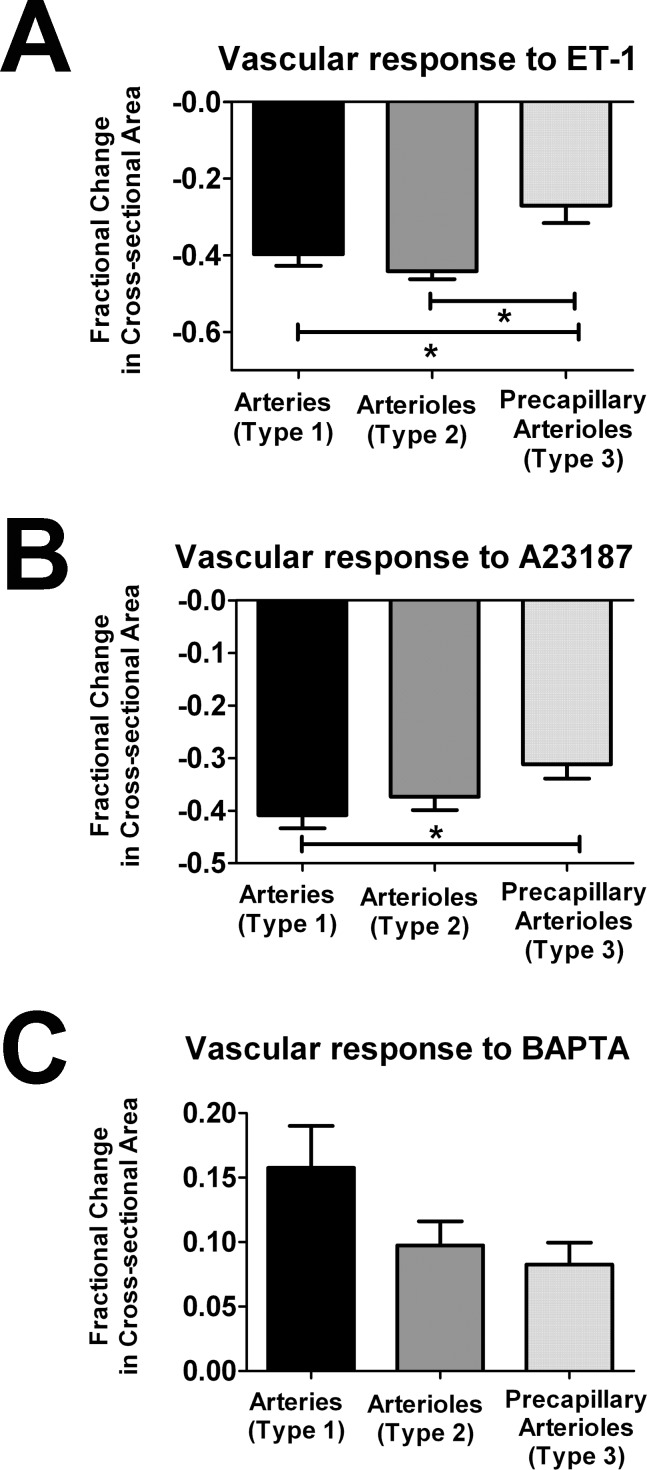
Choroidal vessel responses to applications of vasoconstriction and vasodilation agents according to vessel type. Live confocal imaging in flat-mounted scleroidal tissues was used to monitor responses of choroidal vessels made visible by GFP-expression in perivascular mural cells. Fractional changes in cross-sectional area 15 minutes following the application of vasoconstriction and vasodilation agents were measured. (**A**) Vasoconstriction in response to ET-1 (10 nM) was observed in all vessel types, with Type 3 vessels demonstrating significantly lower fractional vasoconstriction compared to both Type 1 and Type 2 vessels. (**B**) Vasoconstriction in response to A23187 (10 µM), a calcium channel ionophore, was observed in all vessel types, with Type 3 vessels demonstrating significantly lower fractional vasoconstriction compared to Type 1 vessels. (**C**) Vasodilation in response to BAPTA (10 µM), a calcium chelator, was observed in all vessel types. There was a trend of decreasing fractional change with vessels with decreasing density of perivascular mural cells, but these differences did not reach statistical significance. (* indicates p<0.05, 1-way ANOVA using the Kruskal-Wallis test, with the Dunn’s multiple comparison test, n = 8 to 41 measurements from ≥3 biological replicates).

We also compared the fractional changes in cross-sectional area occurring inType 1, 2, and 3 choroidal vessels following induction. All three vessel types responded to ET-1 by constriction but the fractional decrease in cross-sectional area was significantly lower in Type 3 vessels compared to Type 1 and 2 (Fig. 10A). With A23107 application, Type 3 vessels also demonstrated the smallest fractional decrease in cross-sectional area (Fig. 10B). The relative magnitudes of vasodilation in response to BAPTA was also in the order of Type 1>Type 2>Type 3, although these did not reach statistical significance (Fig. 10C).

## Discussion

In this study, we have employed a transgenic mouse model expressing GFP under the control of the α-SMA promoter to characterize the distribution, morphology, and contractility of mural cells at different levels of the choroidal circulation in a live, *ex vivo* tissue preparation. Although this mouse model has been previously used in the *in vivo* imaging of retinal perivascular mural cells [23] and lineage tracing in the bone marrow and kidney [22], a detailed structural and functional examination of corresponding cells in the choroid has not been previously performed. This transgenic mouse model in a non-pigmented background confers a number of technical advantages: (1) the delineation of the entire morphology of the mural cell by the intracytoplasmic distribution of GFP, (2) a clear *en-face* visualization of mural cells throughout the choroid beneath an intact RPE layer, and (3) the ability to examine mural cells in a living state, allowing their behavioral responses to be recorded by dynamic live-cell imaging, absent any potential structural artifacts induced by fixation or tissue processing.

We found in the study that α-SMA-expressing perivascular mural cells in the choroidal circulation demonstrated significant diversity in distribution and morphology at each level of the vasculature and appeared to have differing spatial relationships and extents of physical contact with the underlying endothelial cell layer. These diverse phenotypes can provide insight into the potential functional roles of mural cells which can be addressed in functional studies. Although we have employed the term “mural cell” to refer generally to the GFP-labeled cells in the choroid [26], it is clear that at the level of arteries and primary arterioles they are constituted by smooth muscle cells, while at the level of the precapillary arterioles and the choriocapillaries, they meet the morphologic criteria for pericytes (defined as intramural cells enveloping the endothelial cells of microvessels). We did not observe here any definite demarcation in the transition between smooth muscle cells and pericytes in terms of morphology, density, or immunopositivity for any of the markers used (NG2, desmin, α-SMA). It is likely that smooth muscle cells and pericytes in the choroid represent a continuous spectrum of phenotypic variants that had been derived from a common precursor.

The diversity of mural cell distributions and morphologies described here raises a number of hypotheses concerning their function in the choroid. Pericytes in the vasculature of different organ systems have been described as showing “selective positioning” that may regulate blood flow, maintain vessel integrity, and facilitate vascular transport [27]. One key role proposed for choroidal mural cells involves the regulation of choroidal blood flow through circumferential contractile movements that result in changes in vascular diameter. Physiological regulation of choroidal blood flow has been thought important in controlling the supply of oxygen and nutrients to the outer retina and in thermoregulatory functions [1]. Dysregulation of choroidal blood flow has also been described in retinal pathologies and animal models of disease including diabetic retinopathy [11], [13], [28] and age-related macular degeneration [29]–[32]. In order to carry this role in a regulated manner, the choroid may require a diversified set of contractile perivascular “machinery” to generate optimized blood flow control at different levels of the choroidal circulation. In the current study, we demonstrate that pericytes and smooth muscle cells of the choroid demonstrate contractility resulting in vasoconstriction. While pericyte contractility has been previously studied in cultured pericytes *in vitro* [33], [34], in isolated microvessels [35], as well as in retinal explants and cerebellar slices [21], the existence of contractility in choroidal pericytes has not previously been specifically demonstrated, and has indeed in recent reports been called into question [14], [20]. We also noted that the fractional change in diameter in choroidal vessels induced by contracting mural cells correlated with cell density and morphology – i.e. vessels containing a higher density of mural cells with more circumferential morphologies demonstrated a greater degree of contractility. This indicated that the diverse forms mural cells in the choroid may be patterned and specified to confer different capabilities for vasoregulation at different levels of the choroidal vasculature.

Our live time-lapse recordings of responses of mural cells in the intact choroid to vasoconstrictive agents indicate that contractility in choroidal mural cells *in situ*, like that in dissociated smooth muscle cells and arteriolar fragments *in vitro* [36], [37], is regulated by intracellular calcium concentration. Regulatory mechanisms, such as choroidal innervation, hypercapnia, circulating molecules, and local determinants [1], [38], are likely to converge on the choroidal mural cell to regulate its contractility via processes of calcium entry and release from intracellular stores [37]. In particular, autonomic innervation of the choroid may be constitute a key regulatory mechanism that is not common to the neuroretina as retinal vessels lack a similar innervation [39]–[41]. Mural cells in the choroid have been demonstrated to receive direct autonomic innervation via the perivascular plexus, a dense network of fibers that terminate around choroidal vessels [1]. These include sympathetic and parasympathetic fibers, the latter of which also release somatostatin [42], vasoactive intestinal peptide (VIP) [43] and nitric oxide (NO) as vasoactive agents [44], [45]. Sympathetic and parasympathetic innervation may also influence mural cells indirectly via intrinsic choroidal neurons that are located within the choroid [46], [47] which also terminate on mural cells [48] and use VIP and NO as transmitters [43]. How these patterns of innervation terminate differentially on mural cells at each level of the choroidal vascular tree to regulate mural cell contractility is unknown and constitutes an interesting subject for further investigation. The living sclerochoroidal flat-mount experimental preparation described in this study may amenable to experiments that combine live-cell morphological imaging with fluorescence-based calcium imaging in order to further dissect the effects of these external influences.

In addition to vasoregulation, the diversity of mural cells may confer other functions particular to the choroid. In particular, pericytes in the choriocapillaris having a sparse and non-circumferential distribution appear unlikely to contribute to vasoconstriction. It is intriguing that these cells, whose existence has at times been doubted [26], have a prominently polarized distribution, being completely absent from the vitreal surface of the choriocapillaris and present only on the scleral surface. This arrangement may be an adaptation to avoid physical obstruction of transport between the choriocapillaris and the retina but how the pericytes contribute to choriocapillaris function remains mysterious. It is possible that under normal conditions, they may serve to mediate particular endothelial/pericyte interactions that underlie structural, trophic, or physiological influences [49], [50]. Conversely, the low coverage of pericytes here is an adaptation that allows for the structural plasticity of the vasculature, permitting remodeling in response to changing physiological conditions [51]. However, this adaptation may have deleterious consequences as under pathological conditions as it may predispose the choriocapillaris and terminal choroidal vessels to vascular instability that are permissive to the formation of choroidal neovascularization [14].

In summary, we have employed a versatile and intact choroidal preparation to analyze the distribution, density, and morphological features of perivascular mural cells in the mouse choroid at different levels of the choroidal vasculature, including the choriocapillaris. Using live-imaging techniques, we show that choroidal pericytes and smooth muscle cells demonstrate contractility that results in vasoconstriction, using mechanisms that are regulated by intracellular calcium. The diversity of mural cell distributions and morphologies was correlated with the capability for vasoconstriction, suggesting that mural cells may be regionally specialized depending on the need for blood-flow regulation. These morphological and behavioral characterizations establish a framework for future explorations into the role that perivascular mural cells play in the physiological and pathological functioning of the choroid.

## Supporting Information

Figure S1 **Perivascular mural cells are surrounded by a laminin-positive basement membrane.** Immunohistochemistry for laminin demonstrates that individual perivacular mural cells are surrounded by a laminin-positive basement membrane for type 1 (A–C), type 2, (D–F), and type 3 (G–I) mural cells.
(TIF)

Movie S1 **3-dimensional reconstruction of a confocal z-stack of images taken of the choriocapillaris layer of the choroid.** Choriocapillaries vascular lobules are labeled by DiI-perfusion (red) and associated mural cells are labeled with GFP (green). Rotation of the image reconstruction demonstrates the distribution of mural cells as widely spaced cells with horizontally-oriented proceses which are closely opposed the the scleral surface of the choriocapillaris, in contact with vascular lobules. These mural cells are notably absent from the vitreal surface of the choriocapillaris that opposes Bruch’s membrane.
(AVI)

Movie S2 **Response of a choroidal arteriole (type 2 vessel) before and at different time points after the application of endothelin-1 (ET-1, 10 nM) in the recording medium.** Constriction of the vessel was observed 5 minutes after ET-1 application and sustained for the 15 minutes of ET-1 application. Vascular constriction was long-lasting and only fractionally reversed 60 minutes after washout of ET-1 from the recording chamber.
(AVI)

Movie S3 **Response of a choroidal arteriole (type 3 vessel) before and at different time points after the application of calcium ionophore A23187 (10 µM) in the recording medium.** Constriction of the vessel was observed 5 minutes after A23187application and sustained for the 30 minutes of A23187 application. Vascular constriction was long-lasting and only fractionally reversed 60 minutes after washout.
(AVI)

Movie S4 **Response of a choroidal arteriole (type 2 vessel) before and at different time points after the application of the calcium chelator, BAPTA (10 µM) in the recording medium.** Dilatation of the vessel was observed 5 minutes after BAPTA application and partially reversed 40 minutes after washout from the recording chamber.
(AVI)
