# Supplementary figures and images for: Perivascular Mural Cells of the Mouse Choroid Demonstrate Morphological Diversity That Is Correlated to Vasoregulatory Function
Source: PLoS One. 2013 Jan 4;8(1):e53386. doi: 10.1371/journal.pone.0053386 (PMC3537675; doi:10.1371/journal.pone.0053386)

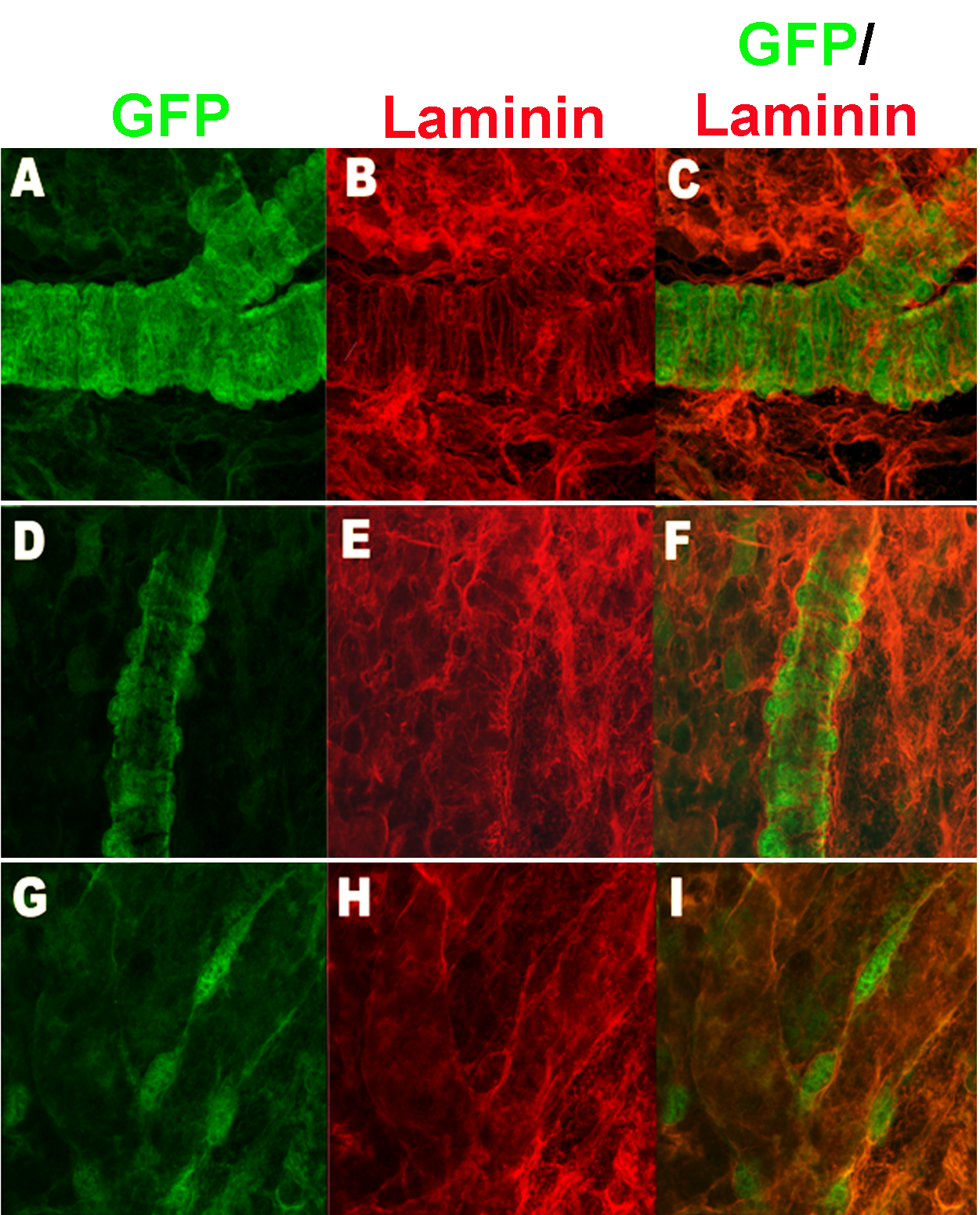

Supplement: Figure S1 — Perivascular mural cells are surrounded by a laminin-positive basement membrane. Immunohistochemistry for laminin demonstrates that individual perivacular mural cells are surrounded by a laminin-positive basement membrane for type 1 (A–C), type 2, (D–F), and type 3 (G–I) mural cells. (TIF) [file pone.0053386.s001.tif]
